# Development and Evaluation of a Decision Aid to Support Patients’ Participatory Decision-Making for Tumor-Specific and Palliative Therapy for Advanced Cancer: Protocol for a Pre-Post Study

**DOI:** 10.2196/24954

**Published:** 2021-09-17

**Authors:** Katsiaryna Laryionava, Jan Schildmann, Michael Wensing, Ullrich Wedding, Bastian Surmann, Lena Woydack, Katja Krug, Eva Winkler

**Affiliations:** 1 Institute for History and Ethics of Medicine Centre for Health Sciences Martin Luther University Halle-Wittenberg Halle (Saale) Germany; 2 Department of Medical Oncology National Center for Tumor Diseases Heidelberg University Hospital Heidelberg Germany; 3 Department of General Practice and Health Services Research Heidelberg University Hospital Heidelberg Germany; 4 Palliative Care Department of Internal Medicine II University of Jena Jena Germany; 5 Health Economics and Health Care Management Bielefeld University Bielefeld Germany

**Keywords:** decision aid, neoplasms, palliative care, clinical trials, longitudinal study

## Abstract

**Background:**

To support advanced cancer patients and their oncologists in therapeutic decisions, we aim to develop a decision aid (DA) in a multiphased, bicentric study. The DA aims to help patients to better understand risks and benefits of the available treatment options including the options of standard palliative care or cancer-specific treatment (ie, off-label drug use within an individual treatment plan).

**Objective:**

This study protocol outlines the development and testing of the DA in a pre-post study targeting a heterogeneous population of advanced cancer patients.

**Methods:**

In the first step, we will assess patients’ information and decisional needs as well as the views of the health care providers regarding the content and implementation of the DA. Through a scoping review, we aim to analyze specific characteristics of the decision-making process and to specify the treatment options, outcomes, and probabilities. An interdisciplinary research group of experts will develop and review the DA. In the second step, testing of the DA (design and field testing) with patients and oncologists will be conducted. As a last step, we will run a pre-post design study with 70 doctor-patient encounters to assess improvements on the primary study outcome: patients’ level of decisional conflict. In addition, the user acceptance of all involved parties will be tested.

**Results:**

Interviews with cancer patients, oncologists, and health care providers (ie, nurses, nutritionists) as well as a literature review from phase I have been completed. The field testing is scheduled for April 2021 to August 2021, with the final revision scheduled for September 2021. The pre-post study of the DA and acceptance testing are scheduled to start in October 2021 and shall be finished in September 2022.

**Conclusions:**

A unique feature of this study is the development of a DA for patients with different types of advanced cancer, which covers a wide range of topics relevant for patients near the end of life such as forgoing cancer-specific therapy and switching to best supportive care.

**Trial Registration:**

ClinicalTrials.gov NCT04606238; https://clinicaltrials.gov/ct2/show/NCT04606238.

**International Registered Report Identifier (IRRID):**

DERR1-10.2196/24954

## Introduction

### Background

Patients with advanced cancer, when most standard therapies have been administered, are confronted with complex decisions about the further course of their treatment. They might face such decisions as (1) forgoing cancer-specific treatment and focusing on best supportive care or (2) off-label treatment within an individual treatment plan or possible inclusion in early clinical trials (ie, phase I and II studies).

Such decisions are complex and require the consideration of patients’ decision-making values and treatment preferences as well as weighing different factors such as patients’ quality of life, therapy side effects, and uncertainty about possible treatment outcomes [1,2]. We use the term decision-making values for the importance that patients place on the options’ positive and negative aspects when considering a specific decision. Treatment preferences refer to the degree to which each patient prefers each treatment option [3]. Treatment preferences (ie, regarding family involvement in decisions or the extent of participation in decision making) and patients’ decision-making values (ie, for quality or length of life) might differ considerably [3]. Current guidelines on advanced care planning [4] emphasize the importance of consideration and timely integration of patients' treatment preferences and decision-making values in decision making in advanced cancer [3,5,6].

Furthermore, compounding the decisional process is that, on the one hand, oncologists often avoid prognosticating and eliciting patients’ treatment preferences for or against anticancer therapy and decision-making values [5-9]; on the other hand, many patients tend to have an inaccurate perception of the curability of their cancer [5,10-13]. A multicenter international study conducted with 1390 patients with advanced cancer demonstrated that 55% of patients receiving palliative care thought that their cancer was curable [10]. The same is true of patients participating in clinical trials. Many of them tend to have therapeutic misconception. They misunderstand a trial’s purpose and express “unrealistic optimism” regarding its benefits [14-18]. A study with 301 cancer patients with gastrointestinal, gynecological, and lung cancers showed that more than 80% of patients in phase 1 clinical trials expected clinical benefits (ie, tumor shrinkage) and 10% even hoped for a cure [18].

Against this background, shared decision making (SDM), an approach based on patients’ engagement in the decisional process, becomes of particular importance in advanced cancer planning [11]. SDM has been increasingly advocated as it elicits patients’ decision-making values and treatment preferences. It permits informing patients about treatment benefits and harms and involving patients more actively in care planning, helping them weigh information based on their preferences [2].

Despite the fact that patients with advanced cancer might differ in their coping with disease [12], need for information, and preferred level of involvement in decisions [13,19], there is an urgent need to facilitate SDM and systematically support patients and oncologists in arriving at evidence-informed and value-congruent decisions as these decisions have a major impact on patients’ last months of life [2].

One of the existing possible ways to facilitate SDM is using patient decision aids (DAs). DAs are tools (pamphlets, videos, web-based or paper-based materials) that aim to help patients to participate in decision making. They provide information about different treatment options and patient-related advantages and disadvantages, help patients clarify their health care goals, and elicit and integrate their decision-making values in the decision-making process [20,21].

Various systematic literature reviews on DAs for people facing treatment or screening decisions demonstrated that the use of DAs can improve SDM, aligning decisions with the preferences of patients without negative impact on clinical outcomes. DAs have been shown to increase patients’ involvement in decision making in various clinical domains as well as to improve patients’ informed choices and to facilitate challenging discussions about goals of care and advanced care planning. Studies show that using DAs can contribute significantly to reducing decisional conflict and make patients feel more confident to make decisions [20,22-24]. The use of DAs has been shown to be associated with patients’ increased knowledge of treatment options [20]. Patients who used DAs had a more accurate risk perception and more realistic expectations of therapy outcomes [18,25,26]. For example, a study conducted with 40 hospitalized patients with advanced cancer demonstrated that a video DA reduced patients’ decisional uncertainty while increasing patients’ knowledge and readiness for palliative radiation therapy [27]. Furthermore, they could contribute to patients’ satisfaction with decisions and minimize patients and caregivers’ regret and blame on physicians [20,26,28-31].

However, many of the available DAs target patients considering therapy for early-stage cancer, cancer screening, or decisions about genetic testing and rarely target patients considering the management of advanced cancer [26,32-35].

There are only a few DAs for advanced cancer patients even though recommendations for their systematic development have been published [21]. Existing DAs are limited in their use by a certain type of cancer (ie, colorectal cancer or prostate cancer [36,37]) or target certain treatment scenarios (ie, participation in early phase clinical trials [2], use of standard systemic cancer therapies [38,39], or initial treatment after diagnoses [40]). To our knowledge, one patient communication aid in the Dutch language has been developed that targets clarification of patients’ preferences and encompasses a question prompt list that can be used by patients with advanced cancer regardless of tumor type when talking to their oncologist [11].

### Study Objectives

Against this background, we aim to develop a patient DA with a patient-centered design to support advanced cancer patients and their oncologists in decision making about anticancer treatment in situations where standard anticancer treatment lines have been exhausted. The DA will be aimed at a heterogeneous population of cancer patients, independently of cancer type as it will focus on situations that are generic for patients when standard treatment options are about to be exhausted. These decisions are typically similar for all cancer types and are about forgoing cancer-specific therapy and switching to best supportive care. The alternative is an additional tumor-specific treatment as off-label treatment, experimental individual treatment plan, or treatment within a clinical trial. With such a generic DA, we aim to facilitate routine initiation of the end-of-life discussions that still occur too late in the course of disease. Second, we aim to develop a clinically feasible implementation plan for the DA.

Furthermore, we aim to assess the acceptance and test potential effects of the developed DA on (1) oncologist-patient interaction (ie, satisfaction with the oncologist-patient interaction), (2) patient involvement in decision making, and (3) level of decisional conflict and uncertainties about choices.

### Conceptional Framework

The novelty of this study and the planned DA is that it targets patients with different types of advanced cancer in order to provide them with information and help them decide about the continuation or forgoing of tumor-specific therapy in clinical situations, in which there is no further standard tumor-specific therapy available but treatment is available as off-label treatment or part of a trial. Hence, the DA aims to facilitate SDM, which is essentially the communication of the best-available research evidence on benefits and harms of options and the clarification of patients’ treatment preferences in relation to this information [41]. Based on a consensus of experts in the field [41], SDM has been defined in terms of 3 broad phases: (1) team talk: work together, describe choices, offer support, and ask about patients’ goals; (2) option talk: discuss alternatives using risk communication principles; and (3) decision talk: get to informed preferences, make preference-based decisions.

The DA in our project is used to influence phase 1 of the SDM, which will probably affect the health care provider-patient communication in phases 2 and 3 as well. Based on the available body of randomized trials on clinical DAs [20], we primarily expect decreased patient decisional conflict and an increased feeling of being informed. Although the impact of DAs on professional performance seems limited overall, the use of the DA with patients with advanced cancer may also reduce intensive treatment. While we will measure quality of life in our study, we do not expect changes as a result of the use of the DA based on the available research evidence [20]. Given the practice-based character of the study, we did not specify and measure psychological factors and processes that may be affected by the DA, such as coping mechanisms.

## Methods

### Overview

We will use a systematic stepwise development process for the DA based on the International Patient Decision Aid Standards (IPDAS) collaboration guidelines, which are particularly relevant to “preference-sensitive” decisions [21,42]. The process will include the following key steps of DA development and testing: (1) establishing the informational basis for patients’ and health care providers’ decisional and information needs, (2) development and review of the DA by the interdisciplinary research group and presenting it in research colloquiums, (3) testing of the DA (design and field testing) with patients and health care providers (understandability, acceptance, usability, and feasibility testing) as well as usage instruction with health care providers, (4) pilot study in a pre-post design with baseline and intervention phases to evaluate the DA with regard to 3 objectives of feasibility and pilot research [43]: testing procedures, estimating recruitment and retention, and determining sample size. The first 2 objectives primarily relate to feasibility, while the third objective relates to the effectiveness of the intervention (the smaller the potential effects, the higher the sample size in a definitive trial needs to be). For the third objective, we have included patient-related outcomes and have planned to explore the potential effects of the DA in a pre-post comparison. The intervention phase will include user acceptance testing with oncologists and patients.

**Figure 1:**
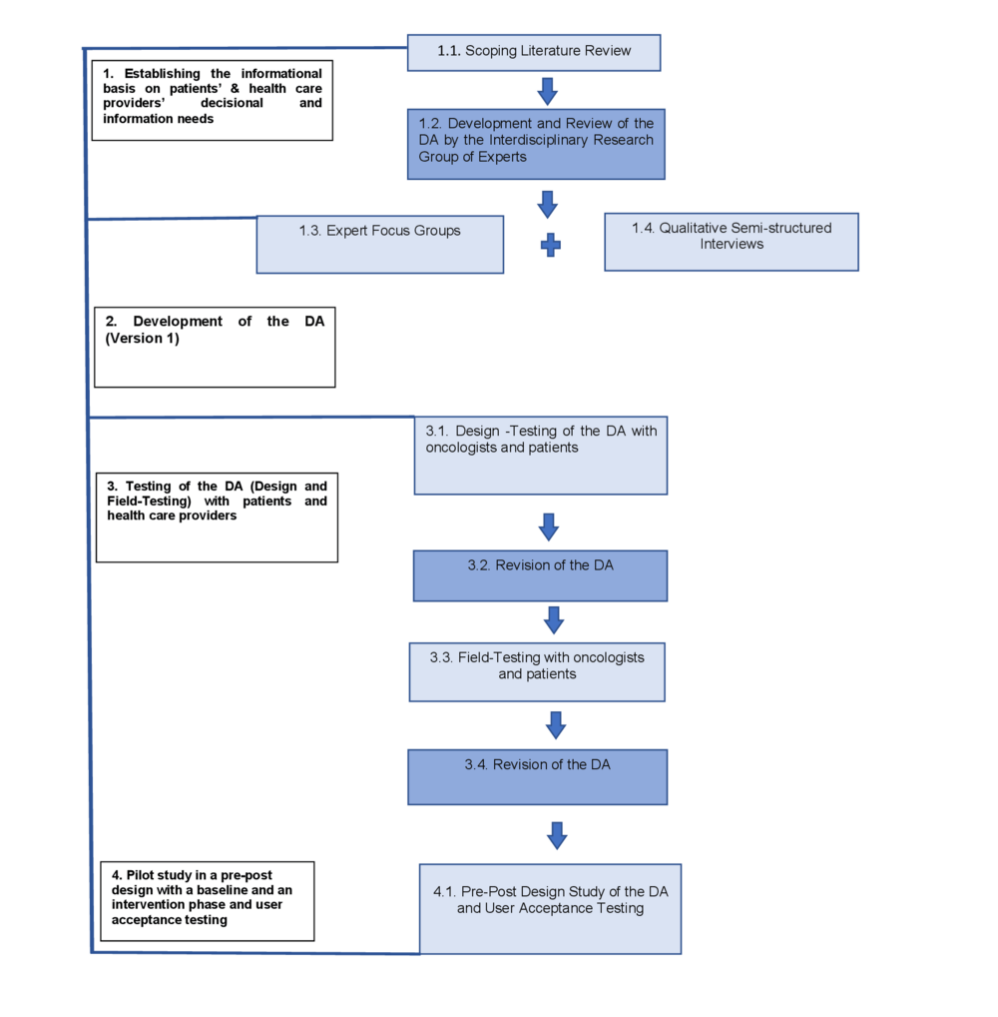
Overview of the decision aid (DA) development process based on the model by Coulter et al [44].

The model of the DA development process based on the model by Coulter et al [44] is presented in Figure 1.

### Study Setting

The study is bicentric and will be carried out at the Department of Medical Oncology, National Center for Tumor Diseases (NCT), Heidelberg University Hospital, Heidelberg, Germany and at the Department of Internal Medicine II (Palliative Care), University of Jena, Jena Germany. Patients will be recruited in Heidelberg (oncology) and Jena (specialized palliative care). This study was approved by the Ethics Committee of the Medical Faculty of the University of Heidelberg and University of Jena.

### Establishing the Informational Basis on Patients’ and Care Providers’ Decisional and Information Needs

As a first step in our study, we plan on assessing patients’ and care providers’ decisional and information needs as well as care providers’ and patients’ decision-making pathways. We aim to analyze specific characteristics of the decision-making process in patients with advanced cancer, to work out the content, features, and best way to deliver the DA.

### Scoping Literature Review

Substantial evidence is already available on the content of decisions made by advanced cancer patients and their caregivers, as well as on their treatment preferences that influence such decisions. In addition, as mentioned earlier, some DAs have already been developed for this context. To provide an overview of the current state of research, we aim to conduct a scoping review as a first step in this study.

The objective of this scoping review is twofold: first, to assess the current state of research literature, as well as the grey literature, on patients and caregivers and their priorities regarding choices on advanced cancer management in the last months of life. This will also involve examining already existing DAs in this particular field. Second, we aim to systematically analyze studies on DAs for patients with advanced cancer in order to gather information on technical issues, such as the design of DAs or information on the facilitating and inhibiting factors in design and implementation. For this purpose, both development and evaluation studies on DAs will be reviewed and their quality assessed using the criteria from the IPDAS Collaboration [21].

The scoping review will be conducted jointly by research associates from the department of Health Economics and Health Care Management of Bielefeld University, Institute for History and Ethics of Medicine of Martin Luther University Halle-Wittenberg, and Department of Medical Oncology of the NCT, to ensure that value-related aspects of decision making will be covered as part of the review. We will search pertinent databases (Medline, PsycInfo, Web of Science, DIMDI, Euroethics) up to December 2019 for relevant publications in the English or German language. Various keywords for synonyms of decision making as well as cancer and end-of-life care will be used to include relevant studies. The findings of this scoping review will be reported according to the PRISMA-ScR (Preferred Reporting Items for Systematic Reviews and Meta-analysis Extension for Scoping Reviews) guidelines [45].

In order to ensure transparency and consistency in the reporting of results, this scoping review will follow the methodological framework for scoping reviews developed by Arksey and O’Malley [46] with the proposed improvements by Levac et al [47] and Peters et al [48]. This framework consists of the following stages: (1) identifying the research question; (2) identifying relevant studies; (3) selecting studies; (4) charting the data; (5) collating, summarizing, and reporting the results; (6) expert consultation.

The insights from this scoping review will inform the subsequent focus groups with clinical experts and semistructured interviews with patients.

### Establishment of the Interdisciplinary Research Group of Experts

The expert group will consist of the team members of our research group (n=4) who will have mostly a supervision function and advisory function. It will include an oncologist, leader of 1 palliative care unit, leader of a medical ethics department, and leader of a department in health service research. Members of this group will not conduct the field research but will serve as active research team members who guide, review, edit, and approve each step of the research process. Their major role is to guide the overall study and to ensure the patient DA will be patient-centered, meaningful, understandable, usable, and feasible for rapid implementation.

### Expert Focus Groups

As a second step, we plan to conduct 3 focus group discussions. A focus group interview is a semistructured discussion with a group of experts where different topics can be explored in participants’ interactions with each other [49]. The successful implementation and follow-up use of the DA depend on oncologists’ willingness to discuss the DA together with the patients during the consultations. Furthermore, based on their experiences, oncologists can shed light on many important aspects of patients’ decision making. Thus, their perspectives seem to be essential for the development of the DA.

The major aim of our focus group interviews is to identify existing difficulties in discussing tumor-specific and palliative therapy with patients, implementation strategies, and possible barriers as well as the best timing for using the DA.

The focus group interviews will be used to generate information on potential factors relevant to the sampling and content of the semistructured face-to face interviews with patients. When planning the focus groups and later reporting on the results, we will refer to the COREQ (consolidated criteria for reporting qualitative research) checklist for in-depth interviews and focus groups that covers all important components of a focus group study design [49].

#### Participants, Setting, and Data Collection

Participants (5-8 persons per focus group) will be recruited at both study sites (Jena and Heidelberg). Three focus groups are planned: 2 focus groups will include oncologists with different levels of working experience, and the other will be conducted with a multidisciplinary team of support services. We plan to include nurses, specialists from an ambulatory palliative care team, nutrition, counseling social work, and psycho-oncology for this focus group. Participants will be purposely sampled to represent different levels of working experience, age, and gender to reflect a wide range of opinions. Participants will be approached by telephone by a study nurse. The focus groups will last approximately 90 minutes (each focus group).

#### Development of the Interview Guide

An interview discussion guide will be developed by a multidisciplinary team including expertise from clinical oncology, social sciences, and medicine ethics and pretested with 2 oncologists and 2 nurses before being used in the subsequent focus groups. The primary results of the literature review will be incorporated into the development of the interview guide. Three major topics for the focus groups will encompass the most important aspects that could be helpful for the development of the DA. Every topic will have several questions in order to gain deep insight into the decision-making process in advanced cancer regarding the context, use, and implementation of DAs. Furthermore, based on the literature review, we decided to develop a preliminary DA prototype in order to discuss it at the end of each focus group. This preliminary prototype will include the most important aspect of a DA and will be used as a trigger for a discussion about the implementation and development of our DA. The aim is to figure out which aspects identified in the literature review might be useful for our DA. An iterative process will be used so that the information derived from the first focus group will be analyzed and the interview guide will be revised and adjusted for the next focus groups.

The interview guide topics are presented in Textbox 1.

**Focus group topics.**

1. Questions on the decision-making process in advanced cancer about:
  limiting cancer-specific treatment, possible off-label drug use, and integration of supportive care
  patients’ inclusion in early clinical studies (important factors for oncologists and patients)
  clinical scenarios in advanced cancer care
2. Questions on the decision aid (DA), including:
  context and form of the DA
  time of use
  implementation barriers and implementation strategy
3. Discussion of the DA prototype (a DA prototype has been developed before the focus group interviews and will be shown at the end of the focus group for discussion)

#### Analysis of the Focus Groups

Interviews will be audiotaped, transcribed verbatim, and analyzed according to qualitative inductive content analysis as described by Sandelowski [50]. We decided to use this approach as there is not much research on DAs that are not limited to a certain cancer type in advanced cancer near the end of life [21,36,37]. As this content might differ considerably from usual cancer DAs, we favored a more open approach without using the pre-existing categories for analysis as suggested by deductive content analysis. Inductive content analysis includes open coding, creating categories, and themes.

In the first step of open coding, interviews will be analyzed “sentence by sentence” while codes will be written in the text. In the next step, the codes will be categorized. After this, the lists of categories will be grouped into themes [51]. The coding will be conducted by 2 researchers (KL and BS) who will discuss coding disagreements and refine the coding system. To minimize the personal bias and reflexivity of 2 single researchers, we plan to discuss results from every coding round in our interdisciplinary research team. Furthermore, the final results and possible input for the development of the DA and for the patients’ interviews will be discussed in our research team. This analysis will be conducted with the help of the data analysis software MAXQDA (VERBI GmbH, Berlin, Germany).

### Qualitative Semistructured Interviews

The aim of qualitative interviews is the assessment of patients’ decision-making values and treatment preferences associated with the decision-making process when standard systemic therapies have been exhausted, as well as factors contributing to the decisional conflict. As the interview topics are about forgoing cancer-specific treatment and are very sensitive for patients, a qualitative approach has been selected as it mostly suited for exploring sensitive topics [52]. In spite of the fact that family members play an important role in decision making in advanced cancer, we decided to exclude them from interviews due to difficulties in the recruitment process.

### Participants, Setting, and Data Collection

Patients will be recruited at the NCT (University Hospital Heidelberg) in the in- and outpatient settings. We will include adult patients with incurable, stage IV disease (prostate, breast, pancreatic, stomach, or colorectal cancer) in an advanced treatment stage (prognosis <12 months and/or standard palliative care only is considered). These entities cover a large proportion of cancer burden. In addition, these are the entities covered by the outpatient clinics of the Department of Medical Oncology of the NCT. These inclusion criteria make it possible to interview patient groups who are potential users of a DA at an advanced cancer stage when decisions about forgoing cancer-specific treatment are usually discussed with patients. Furthermore, we will try to include patients who have already made at least 1 of the 3 decisions. However, the focus will be on the orientation towards the physical and mental condition of patients. An experienced oncologist will be in charge for the recruitment and will decide if patients’ psychological states will allow them to participate in the interview. If a patient has physical symptoms (ie, pain) or his or her physical condition worsens, he or she will not be asked to participate in the interview. Included patients will be monitored by their oncologists after the interview, and if necessary, in case of distress, a psycho-oncology team will be contacted to support the patient. Patients must have an adequate level of the German language and be willing and able to give informed consent for participation in the study.

Patients that already are under standard palliative care only, are cognitively impaired, have extreme anxiety or distress, or have a severe comorbid illness, excluding antitumor treatment as assessed by the treating oncologist, will be excluded from the study.

Interviews will be conducted until informational saturation will be reached. We will transcribe and code interviews one by one in order to control the process data acquisition and if necessary, we will adjust the interview guide. Furthermore, this will allow us to control whether saturation has been reached. Regarding saturation, we understand this as the point in our research process when little or no new information emerge in from the data collection and analysis [53]. We opted for this prolonged process as the research is conducted with a very vulnerable patient group who are near the end of life and who have different health conditions.

Thereby, we aim to maximize variation in the purposeful sampling, considering relevant factors that might have an impact on interview results: age, level of education, gender, residential environment (eg, living alone), physical distress related to prior treatment, treatment duration, and availability of experimental treatment. A minimum number of 20 interviews will be performed to address the likely heterogeneity of the underlying cancer entities.

### Development of the Interview Guide

Based on the results of the scoping review and focus groups, the semistructured interview guide will be developed jointly with the researcher of medical ethics and health economics and health care management and involve representatives from oncology to ensure that ethically relevant as well as clinical aspects will be included. As this patient group is very vulnerable due to their far advanced cancer disease and being near the end of life, the interview needs a sensitive approach. We will work with a case scenario in which a similar treatment situation for an advanced cancer patient will be described. Working with a case scenario will help to avoid confronting patients directly with sensitive questions that they might not feel comfortable disclosing or might even escape their own awareness but at the same time will help to explore patients’ treatment preferences as well as knowledge about decision alternatives and corresponding values. For practical reasons, we decided to use only one case scenario in order not to burden patients with interviews that are too long. The wording will be as neutral as possible in order to avoid potential influences on the patients’ thinking processes. We aim to use the case scenario as an entry point into the interview and then ask the patients about their own decision making if they demonstrate a willingness to do so during the interview.

The case scenario and the interview topics are presented in Textbox 2. The questions for the interviews will be formulated to ensure patient-centered and literary-sensitive language and will be pretested in pilot interviews with some patients (n=3). After a test with patients and discussion of the results in our expert groups, the interview guide will be improved so that the interview guide can be elaborated based on the patients’ viewpoints and experiences. Trained research personnel in social sciences and medical ethics will conduct the face-to-face interviews. The interviews will last approximately 30-60 minutes each.

**Topics for patients’ interviews.**

Case scenario about an advanced cancer patient facing decision of treatment limitation: "A 64-year-old female patient received a diagnosis of colorectal cancer with metastasis 4 years ago. The tumor was operated but, in some time, came back. At the moment — like nearly continuously for 3.5 years — she has been treated with chemotherapy. Now the patient has found out that the cancer has gotten worse and that the therapy is not effective anymore. Further cancer-specific treatment could be off-label drug use within an individual treatment plan or inclusion of the patient in an early clinical trial, where a new drug will be tested. However, these treatment options have uncertain outcomes and unclear risks."

There are the following possible scenarios for this patient: (1) forgoing cancer-specific treatment and switching to standard palliative care or (2) individual off-label drug use or inclusion in a clinical trial.

1. Questions on this case scenario regarding the decision-making process about forgoing cancer-specific therapy (ie, If you put yourself in the position of the patient, what information would you need to make a decision?)
2. Questions on patients’ treatment preferences and decision-making values
3. Questions on the decision aid: context, form, and time of use of a decision aid

### Analysis of Qualitative Interviews

The analysis of the qualitative interviews will be conducted analogous to the focus groups (see Analysis of the Focus Groups).

### Development of a DA (Version 1)

Based on the scoping literature review, expert focus groups, and qualitative semistructured interviews, a prototype version of the DA will be developed following the criteria for developing DAs provided by the IPDAS [21]: assessment of users’ needs to discuss options (results of the literature review, focus groups, and interviews), presenting information in a balanced manner, using plain language (design testing), and field testing [21]. It should be emphasized that deviations from the IPDAS could be possible following the qualitative part of our study.

We anticipate that the DA will encompass treatment options (cancer-specific treatment [ie, off-label use of therapies] vs standard palliative care) with risks and benefits as well as preferences and other factors that are associated with decisions in advanced cancer care. 

Due to the proposed generic use of the DA (various tumor entities with a respective variance of options), the rapid development of therapies, and evolving clinical and scientific knowledge, we refrained from providing explicit evidence in the DA. The prognostic information will be added personally by oncologists during the patient-oncologist consultation. To guide the decision conversation and allow for an individually tailored approach, the preference of patients about how much information they like to receive and how much they like to be involved in the decision is assessed as part of the DA.

We are planning a paper-based DA that is introduced to the patient and his or her relatives by an oncologist at the moment that a decision needs to made for or against (continued) oncological treatment in the trajectory of advanced cancer patients. This first draft will be reviewed by the steering group, consisting of 3-5 experts from oncology, medical ethics, and the social sciences to assess the DA in terms of its content and design.

Furthermore, we plan to develop a usage instruction for oncologists. It aims to give indications for oncologists about the timing for DA use, discussion of the DA with patients, and the documentation of the discussion with patients.

### Testing of the DA (Design and Field Testing) With Patients and Health Care Providers

The testing of the DA will include 2 steps. In the first step, we will test the design of the DA with potential users in a controlled environment. In the second step, the DA will be field-tested with patients and oncologists in a real clinical situation, and we will focus on confirming the feasibility of the DA as used in clinical practice. Additionally, the DA usage instruction will be tested with oncologists.

### Design Testing of the DA

In the second project phase, the developed prototype of the DA will undergo a design test. The aim of this testing is to assess the understandability, acceptability, feasibility, and attractiveness of the prototype. Testing will be conducted using face-to face interviews with oncologists (at least 2-3) and cancer patients (at least 6-8) who have been faced with the decision of continuing tumor-specific therapy in the past. Using a think-aloud technique [54] with participants going through the DA as if in the actual decision situation, information about comprehensibility of terms and diagrams used, attractiveness, and manageability of the DA will be elicited from participants’ reactions. Additional questions cover the participants’ opinions of the length, design, and understandability as well as the content of the DA. Moreover, participants will be asked to provide recommendations for possible improvements of the DA. The interview will last approximately 20-30 minutes. Interviews are conducted in the preferred place of the participants (eg, clinic, home) to ensure a comfortable environment where participants can concentrate on the task. Interviews are conducted once with each participant.

### Revision of the DA

Based on the results obtained in the alpha testing, we aim to optimize the prototype draft. After every 2-3 interviews, the DA will be updated based on the feedback of the participants. Alpha testing is completed if there are no further adaptations necessary.

### Field Testing

In the field-testing phase, the feasibility of the DA will be evaluated in “real-world” settings by 16-20 patients and 4-6 oncologists who were not involved in the design phase. Feasibility is operationalized in terms of (1) time required for use and (2) acceptability for patients and oncologists. We will conduct qualitative interviews with patients and oncologists using the following questions:

- What is the best time to use this DA?
- What is the best way to use the DA by oncologists and patients?
- Was the DA helpful to make a treatment decision?
- What did you find good about the DA?
- How do you think the DA could be improved?
- What impact did the DA have on the length of the consultation?
- Would you recommend using this DA? (patients); When would you use the DA in the future? (oncologists)

Each interview will last approximately 20-30 minutes. Qualitative data will be thematically analyzed. The analysis will be conducted analogous to the qualitative analyses described in the qualitative interviews and focus groups.

### Revision of the DA

After every 3-4 interviews, the DA will be revised based on the feedback from the participants. Additionally, relevant observations and feedback are summarized in a guide for use. If necessary, another round of field testing will be conducted. Field testing is completed if, based on the feedback after at least 3 rounds, there are no further adaptations of the DA necessary. Based on the results from field testing, the DA will be revised once again by an expert group, and a final version will be developed.

### Pre-Post Study of the DA and its Acceptance Testing

In the last project phase, we will run a pre-post study of the DA. It has 2 measurement phases: baseline and intervention. The aim of this study is to examine the potential effects of the DA on patients’ knowledge, behavioral changes, and clinical outcomes and to test its acceptance. The Medical Research Council Framework for developing and evaluating complex interventions, which has guided our study, specifies 3 objectives for feasibility and pilot research [43]: (1) testing procedures, (2) estimating recruitment and retention, and (3) determining the sample size. The first 2 objectives primarily relate to feasibility, while the third objective relates to the effectiveness of the intervention (the smaller the potential effects, the higher the sample size needs to be in a definitive trial). For the third objective, we have included patient-related outcomes and plan to explore the potential effects of the DA in a pre-post comparison.

### Design and Settings

First, patients will be recruited in a baseline phase lasting 4 months to observe usual care without using the DA. It means that oncologists will inform patients about further treatment options for cancer-specific therapy, side effects, and potential benefit, but they will not explicitly address that best supportive care would also be an option and will not use a decision support tool for eliciting patient preferences and assisting in decision making. The baseline phase will be followed by an intervention phase lasting 6 months. Oncologists and patients will use the DA in the same situation (change of treatment needs to be discussed with the patient because of disease progression, treatment toxicity, or other reasons). A minimum of 40 doctor-patient encounters will be included in the 4-month baseline, and 40 doctor-patient encounters will also be included in the 6-month DA intervention phase. The whole sample will encompass 80 doctor-patient encounters. The planned sample size is largely determined by the feasibility of recruiting patients within the available project duration.

### Measurements

The potential effect of the DA will be evaluated by testing the improvements on the primary outcome for our study level of decisional conflict. Level of decisional conflict will be measured with the Decision Conflict Scale [55] that assesses patients’ perceptions of uncertainty, modifiable factors contributing to uncertainty, and ultimate satisfaction with the choice. It is one of the most robust and validated instruments to test the impact of DAs in end-of-life decision making [56]; We assume that the Decisional Conflict Scale score will decrease for the patients who use the DA.

Furthermore, we aim to assess the patients’ involvement in decision making, certainty about choice, and satisfaction with the oncologist-patient interaction as exploratory endpoints. Patients’ involvement in decision making will be assessed with the German questionnaire on shared decision making (PEF-FB-9) [57]. The trade-off between patients’ preferences for quality and length of life will be assessed with the validated German version of the Quality-Quantity Questionnaire [1]. The preferred role of the patient in decision making will be assessed with the German version of the Control Preference Scale [58]. Satisfaction with the oncologist-patient interaction will be assessed using the validated Questionnaire on the Quality of Physician-Patient Interaction (QQPPI) [59]. Effect on hope (German version of the Herth Hope Index [60]), anxiety (EQ-5D-5L [61]), quality of life (EORTC QLQ-C30 [62]), and documentation of patient preferences will be measured.

Description of the measures and their psychometric properties, scoring, and interpretation are provided in Table 1.

Such determinants of quality of palliative care as time of integration of specialized palliative services into care, aggressiveness of therapy (anticancer treatment <14 days before death), and place of death will be recorded.

During data collection, patients might have changes in mental status (ie, develop depression or cognitive problems) as well as experience changes in decision making. The study physician involved with data collection will document all possibly relevant factors.

The questionnaires will be completed by patients and oncologists before and after the intervention and will take approximately 15-20 minutes to complete. All assessments will be conducted in German.

**Table 1:** Description of the measures and their psychometric properties, scoring, and interpretation.

Outcome	Instrument	Scoring and interpretation
Level of decisional conflict	Decision Conflict Scale	The German version of the Decision Conflict Scale demonstrated good psychometric properties. The internal consistency was found to be high (Cronbach=.96). It has 5 subscales with a total of 16 items and 5 response categories, ranging from 0 (strongly agree) to 4 (strongly disagree). The total score is calculated in the following way: The 16 Items are (1) summed, (2) divided by 16, and (3) multiplied by 25. Scores range from 0, no decisional conflict, to 100, extremely high decisional conflict.
Patients' involvement in decision making	Questionnaire for participatory decision making (PEF-FB-9)	The German questionnaire on shared decision making “Der Fragebogen zur Partizipativen Entscheidungsfindung” (PEF-FB-9) demonstrated high internal consistency (Cronbach=.93). It has 9 items scored on a 6-point Likert scale, ranging from 0 (not at all) to 6 (fully correct). The score is created by adding all items (range 0-45 points). A higher score means more shared decision making.
Trade-off between patients' preferences for quality and length of life	Quality-Quantity Questionnaire	The validated German version of the Quality-Quantity Questionnaire consists of 9 items in 2 preference dimensions: Q(uality) of life (QL) and L(ength) of life (LL). The scales demonstrated good and acceptable internal consistency (Cronbach=0.71 for LL and .59 for QL). Patients indicate how strongly they agree or disagree with the statements on a 5-point Likert scale. High scores on the quantity or quality scale indicate the importance of the length or quality of life, respectively.
Preferred role of the patient in decision making	Control Preference Scale (CPS)	The preferred role of the patient in decision making will be assessed with the German version of the CPS. It is a valid and reliable measure of preferred roles in medical decision making. It consists of 5 statements (A, B, C, D, E) that each portrays a different role in treatment decision making. For analysis, a categorical variable, which is the person's most preferred role in treatment decision making, will be created. Preference orders will be reclassified into Active (A, B), Collaborative (C), and Passive (D, E).
Satisfaction with the oncologist-patient interaction	Quality of Physician-Patient Interaction (QQPPI)	The German version of the validated QQPPI — “Fragebogen zur Arzt-Patienten-Interaktion” (FAPI) — showed very good reliability (Cronbach=.97). It has 14 items rated on a 5-point scale (range: 1 [I do not agree] to 5 [I fully agree]). The total score is calculated as a mean of all 14 items. The lowest score (1) indicates the lowest quality of physician-patient interaction, and the highest (14) indicates the highest quality of physician-patient interaction.
Effect on hope	German version of the Herth Hope Index (HHI-D)	The HHI-D has satisfactory reliability (Cronbach=.82). It has 12 items rated on a 4-point Likert scale that ranges from 1 (strongly disagree) to 4 (strongly agree), with items #3 and #6 reverse-coded. The scale has 1 global score that ranges from 12 to 48. Higher scores indicate more hope.
Effect on patients' quality of life	EORTC QLQ-C30	The EORTC QLQ-C30 questionnaire is widely used to measure the quality of life of cancer patients. The QLQ-C30 has a global health status scale, 5 functional scales, and 3 symptom scales. High scores on the functional scales mean healthy functioning. A high score for global health status means a higher quality of life. A high score on the symptom scales indicates a high level of problems. Scores for all scales and single items range from 0 to 100. The questionnaires will be interpreted following the official guidelines of the EORTC.
Effect on patients’ anxiety	Anxiety EQ-5D-5L	The EQ-5D-5L, a preference-based measure of general health status, consists of 5 dimensions (mobility, self-care, usual activities, pain/discomfort, and anxiety/depression), each with 5 levels of problems: 1=no, 2=slight, 3=moderate, 4=severe, and 5=extreme or unable to perform the task. The anxiety/depression dimension of the EQ-5D-5L will be used to detect anxiety and depressive symptoms.

### User Acceptance Testing With Oncologists and Patients

A variety of factors can affect users’ acceptance of a DA. Among these factors, users’ perceptions and expectations are the key factors that influence their acceptance. Therefore, we aim to survey the oncologists’ and patients’ acceptance of the DA. The acceptance testing will be conducted during the intervention phase with patients and oncologists who will be caring for the patients in the pre-post study. The major aim is to assess oncologists’ perceptions of the usefulness (how useful oncologists find the DA), willingness to use the DA in daily routine with patients (how comfortable they would be to use the DA with patients), willingness or readiness to use it in clinical practice (how likely they would use it within the next 5 months), and perceived need for the DA. In addition, the patients’ perceptions of usefulness and willingness to use the DA during consultations with their oncologists will be assessed. The oncologists who were involved in the development of DA will be excluded from acceptance testing.

### Measurements

In order to access oncologists’ acceptance of the DA, we plan to use the German version of the Ottawa Acceptability of Decision Rules Instrument (OADRI) [63]. It is a 12-item instrument developed to measure the acceptability of and willingness to use clinical decision rules in the future [64].

Patients’ acceptability will be measured with a question (whether the patient found the DA useful for their decisions) from the DA feedback questionnaire developed by Juraskova et al [65]. Our research team will translate it into German. We refrain from using long questionnaires for patients as they already have to fulfill the set of questionnaires from the pre-post design study.

### Sample Characteristics

Patients will be recruited at participating study sites (Jena, Heidelberg) in inpatient and outpatient settings. Patients will be identified by a study nurse via the electronic health record of patients scheduled in the outpatient or inpatient clinic and approached for participation if they fulfill the inclusion criteria.

We will include adult patients with incurable, stage IV disease (prostate, breast, pancreatic, stomach, or colorectal cancer) in an advanced treatment stage (prognosis <12 months or standard palliative care only is considered). The patient must have an adequate level of the German language to complete the questionnaire and is willing and able to give informed consent for participation in the study.

We will exclude patients from the study who are already under standard palliative care only, are cognitively impaired, have extreme anxiety or distress, or have a severe comorbid illness, excluding antitumor treatment as assessed by the treating oncologist.

The influence of implicit bias will be reduced by recruitment of patients both in Heidelberg and Jena. In addition, the use of a trained study nurse at both sites will reduce implicit bias in recruitment. Drop-out reasons will be documented.

Oncologists will be recruited from the participating study sites (Jena, Heidelberg). They will be identified by a study nurse and invited to participate in the study via phone. We will include oncologists with different levels of working experience and of varying ages. The oncologists who participated in the development of the DA will be excluded from participation in the study.

### Data Analysis

Analyses will be performed using SPSS v.20.0. A descriptive analysis of the sample will be performed. According to the level of variables, means, SDs, medians, minimum and maximum, and absolute or relative frequencies will be reported.

Clinically significant decisional conflict will be calculated and defined by a score of ≥25/100 on the Decision Conflict Scale, which is the most commonly used threshold to distinguish a harmful level of decisional conflict [62-64].

We will compare primary and exploratory outcome measures between the 2 patient groups (baseline and intervention) using Student *t* tests or analysis of variance. Parametric or nonparametric statistics (depending on the distribution of the data) will be used for the analysis of the patient groups from the baseline and intervention phases.

We will compare clinical confounders (time of integration of specialized palliative services into care; aggressiveness of therapy [anticancer treatment <14 days before death and place of death]) between the 2 patient groups (baseline and intervention) by comparing mean values. Measures of variability (SD) will be calculated.

Given the practice-based character and the small sample size of the study, we refrain from extensive measurement and multivariate analysis of mediators and moderators of the intervention effects. For the latter reason, we do not plan to use multiple imputation of missing values. Missing values will be handled by allowing a maximum of 35% missing in the calculation of scale scores.

*P* values of the corresponding statistical tests comparing treatment groups (ie, 2-sample *t* tests for continuous variables and chi-square tests for categorical data) will be given. Statistical significance will be assessed at the level of α=.05 (two-sided).

### Timeline of the Study

The study will be conducted over a period of 3 years, with 12 months for conducting focus groups, interviews, and developing the first version of the DA. In a further 12 months, DA testing will follow as well as the completion of the DA. In the last 12 months of the study, a pre-post study and acceptance analysis will be conducted.

## Results

We anticipate that this study will provide evidence about the developmental process, acceptance, and potential effects of a DA to support advanced cancer patients’ decision making in relation to the limitation of cancer-specific treatment near the end of life. The results will be disseminated through publications in peer-reviewed scientific journals and via presentations at academic conferences. The scoping literature review will provide information on the possible content, features, structure of DAs, and information on fostering and hindering factors in design and implementation strategies. The focus groups will identify factors influencing the decision-making process in advanced cancer, timing, and possible ways of delivery of the DA. Interviews with patients will provide information about patients’ decisional and informational needs, important factors for the decision-making process, and patient preferences and wishes regarding a DA.

Design and field testing will produce an optimized prototype of the DA for further testing. The pre-post study will test the potential effects of the DA on patients’ knowledge, behavioral changes, and clinical outcomes as well as its acceptance.

The field testing is scheduled for April 2021 to August 2021, with the final revision scheduled for September 2021. The pre-post study of the DA and acceptance testing is scheduled to start in October 2021 and shall be finished in September 2022.

## Discussion

The key strength of this study is that it aims at developing a DA that can be effectively used across different types of cancer. Furthermore, a wide range of topics relevant for advanced cancer near the end of life such as forgoing cancer-specific therapy and patients’ inclusion in clinical trials will be covered. To our knowledge, it will be the first DA of its kind.

In trying to develop a DA that is suitable for patients with any cancer type, this certainly will create a number of challenges (in terms of synthesizing the evidence on advanced cancer treatment options and presenting the benefits and risks and choices available). To face this challenge, 2 extensive validation validity phases will be included (review and re-draft of prototype versions 2 and 3), during which the DA will undergo review from advanced cancer patients, oncologists, and other experts from medical ethics and social sciences.

A further strength of this study is that we do not focus solely on the development of a DA but also on testing its impact on clinical practice and on patients’ health outcomes. Additionally, we will evaluate the acceptance of the DA within the implementation study.

Furthermore, for all phases of the DA development and testing, interdisciplinary experts from psycho-oncology, sociology, oncology, palliative medicine, and medical ethics will be involved, enabling a better framework for this process.

This study has several limitations. One of the limitations is that developing and subsequent testing of the DA will take place mainly at the NCT in Heidelberg. The NCT is a national innovative tumor center, combining patient-oriented research, care, and a multidisciplinary approach. It offers a wide spectrum of consulting services for patients such as nutrition, psycho-oncology, pain therapy, pastoral care, self-help, social service, sports, and physical activity. It means that patients have additional services and a more individualized care approach in comparison to other hospitals.

Although the perspective of another hospital (palliative care unit in Jena) will be taken into consideration, it is likely that future multicentric research evaluating our DA’s impact on patients’ outcomes as well as process evaluation of the DA implementation may therefore also be needed.

Furthermore, due to recruitment difficulties, we decided not to interview patients’ family members, who usually are involved in these decisions. Missing this important perspective might have an impact on the results of our study.

In patient interviews, we used a case scenario that might increase social matching bias. Furthermore, there can be a difference between what people think they would do in a described situation and their actual behavior. Furthermore, the interview guide had to be modified and customized according to the patients’ current physical and mental status. This could lead to decreased generalizability of the results.

Our sample size in the pre-post study is relatively small due to the severe conditions of the patients who are near the end of life. This leads to difficulties in recruitment and can restrict the interpretation of the results.

Furthermore, given the relatively small sample size of the planned study, we can only explore the diversity of patients and health care professionals to a specific degree.

## Abbreviations

COREQ: consolidated criteria for reporting qualitative research
DA: decision aid
IPDAS: International Patient Decision Aid Standards
NCT: National Center of Tumor Diseases
OADRI: Ottawa Acceptability of Decision Rules Instrument
PRISMA-ScR: Preferred Reporting Items for Systematic Reviews and Meta-analysis Extension for Scoping Reviews
QQPPI: Questionnaire on the Quality of Physician-Patient Interaction
SDM: shared decision making
